# A Neural Network-Inspired Approach for Improved and True Movie Recommendations

**DOI:** 10.1155/2019/4589060

**Published:** 2019-08-04

**Authors:** Muhammad Ibrahim, Imran Sarwar Bajwa, Riaz Ul-Amin, Bakhtiar Kasi

**Affiliations:** ^1^Department of Computer Science & IT, Islamia University of Bahawalpur, Bahawalpur, Pakistan; ^2^Faculty of Information and Communication Technologies, BUITEMS, Quetta, Pakistan

## Abstract

In the last decade, sentiment analysis, opinion mining, and subjectivity of microblogs in social media have attracted a great deal of attention of researchers. Movie recommendation systems are the tools, which provide valuable services to the users. The data available online are growing gradually because the online activities of users or viewers are increasing day by day. Because of this, big data, analytics, and computational issues have raised. Therefore, we have to improve recommendations services upon the traditional one to make the recommendation system significant and efficient. This article presents the solution for these issues by producing the significant and efficient recommendation services using multivariates (ratings, votes, Twitter likes, and reviews) of movies from multiple external resources which are fetched by the web bot and managed by the Apache Hadoop framework in a distributed manner. Reviews are analyzed by a deep semantic analyzer based on the recurrent neural network (RNN/LSTM attention) with user movie attention (UMA) to produce the emotion. The proposed recommender evaluates multivariates and produces a more significant movie recommendation list according to the taste of the user on a mobile app in an efficient way.

## 1. Introduction

“Recommendation systems” are services that use Artificial Intelligence (AI) and Natural Language Processing (NLP) techniques to provide the empirical solutions of the recommendations for various application frameworks and services [1]. Recommendation systems enables mobile apps and web applications to make the perception intelligently about the selection of different items, movies [2], hotels [3], food [4], tourism [5], books [6], TV shows [7], YouTube videos [8], health [9], etc. Community trends polarize towards music, movies, or videos. For music or movies or videos, a huge amount of stream is available online, but which one of them will be watched is still a rising question. Music or movie recommendation systems still have challenges like the playlist, magnitude, security, privacy, recommendation, and session. Therefore, MRSs become a domain of music information retrieval (MIR) [10–13]. Now, the society has changed, and community trends highly depend on mobile app usage. Several products are enriched by the usage of a mobile app. So mobile app recommendation systems are essential for suitable selection of recommended items [14–16]. Most of the recommender systems are univariate and use ratings and reviews or tweets [17], and other few are bivariate (sentiment score and likes) [18–20]. This work is state of the art and uses the multivariate matrix, which makes the decision using a dynamic approach for suggesting the movie according to the relative taste of the users. The term “multivariate” means involving many variables like a qualitative variable (semantic score) and quantitative variables (Twitter likes, rating, and votes) of movies from three movie sites for significant recommendation [21]. Our work is on extremity grouping of movie reviews, where an opinionated report is labeled with semantic emotions of the microblog text or reviews and emotions [22] using a semantic parser based on the recurrent neural network (RNN/LSTM) [23, 24]. A drawback is that change of a user's review about a movie may affect the user's preference. The nature of reviews influenced by the choice of words uses multilingual dictionaries. Some recommendation systems use linked movie databases, including Trovacinema, Google Places, and Netflix, and Wikipedia provides linked data and ontologies for descriptions about the movie [25–27]. Using the shallow machine learning models for solving the NLP problems is handcrafted and time-consuming. Nowadays, word embedding, neural-based models achieve success and popularity by producing a better result as compared to traditional machine learning logistic regression, SVM, and KNN.

Artificial neural networks are the mathematical models that are inspired by human neural networks. They have three simple layers: input, output, and hidden layers, or sometimes only two layers: input and output layers. The input layer is connected to the hidden layer via a lean weight. The hidden layer output combines via the activation function *h*=*ϕ*(*w*_*i*_ · *x*_*i*_). In the ANN, like the biological neural network, neurons are the nodes, while synopses are the edges. Each artificial neuron has an activation function in the ANN. There are several activation functions like sigmoid which ranges from 0 to 1, hyperbolic function which ranges from −1 to 1, and softmax function whose output in categorical distribution and ReLu function is a feedforward neural network. The ANN is not an algorithm; it is a framework for several machine learning algorithms to solve a complex work. Therefore, we can say that it is a collection of neurons or networks of neurons (https://en.wikipedia.org/wiki/Artificial_neural_network). The recurrent neural network (RNN/LSTM) processes the sequence semantically, which is the basic structure of deep neural networks. Several NLP tasks are performed by RNNs/LSTM attention. In this work, we used the hierarchical neural network (HNN) based on LSTM attention, which impaled the global user and movie information via word and sentence-level attention for document representation. The user's reviews and movie features at the word and sentence level are taken for semantic analysis of reviews, which play a major role in the process of true recommendations. Global user information represents the personal behavior and the movie feature represents a movie genre or a movie profile or linked data which are useful for semantic extraction of movie reviews [28]. In natural language (word sequence), each word or sentence is related to another one and requires to be understood semantically. A huge amount of data are available online on web contents (ratings, reviews, likes, votes, smiley, images, and stars) that can be fetched by a web bot or web agent or crawler, which are all same terms used interchangeably. Web content (ratings, reviews, likes, votes, smiley, images, and stars) is useful for recommendation services. These contents are evaluated and make the perception about users, and items make the recommendation for others [29, 30]. The hot issues of big data like computational complexity are managed by using Map-Reduce and Apache Mahout in NoSQL [31, 32] distributed environment which reduce computation complexity by clustering and horizontal scaling instead of empowered single machine [33]. Because user frequency and data volume gradually increase, it is difficult to manage these huge data by a single machine. Sparsity can be reduced by factorization [34]. Movie recommendation systems provide services to users using content-based filtering algorithms [35], collaborative filtering [36], and some combined forms to make a hybrid filtering algorithm [37]. We used implicate rating to handle the cold start problem [38], an implication managed by the server The multivariate movie recommender provides the services to users to watch the movies according to their profile or history (previously watched or rated). Therefore, there is a need to improve recommendation systems for significant recommendation services. We developed a pilot version for these problems, which consists of a mobile app, a web scraper, and a multivariate recommender to provide the significant services for movie recommendation in an efficient way.

This work is arranged as follows: related works are discussed in Section 2, the recurrent multivariate movie recommendation system model is explained in Section 3, recurrent multivariate movie recommendation system implementation is given in Section 4, experiments and results are discussed in Section 5, and evaluation of the system is done in Section 6. The conclusion of this paper is presented in Section 7 and future work with more parameters in Section 8.

## 2. Literature Review

Sentiment analysis deals with the user's comments, reviews, likeness, ratings, etc. to retrieve the sentiment and opinions of users. The microblog text sentiment analysis is based on the NLP methodology to retrieve suitable YouTube videos and movies and campaigns for smoking cessation, pharmacovigilance, politics of elections, advertisement of pizza, journalistic inquiry, and influenza prevention for public health [39–45]. The CNN and RNN are two major categories of deep neural networks (DNNs). Sequential and hierarchal structures deal with the RNN and CNN, respectively. Both the CNN and RNN can be supervised, semisupervised, and unsupervised. The deep learning algorithm also involves in propagation and weight update activities. RNNs are based on multiple layers: input, hidden, and output layers, while CNNs have input, hidden, and pooling layers. The CNN is efficient for pattern recognition in hierarchal data classification. However, the RNN deals with linear data to be semantically analyzed and classified in NLP; in the CNN, the window size is limited, so the RNN is very useful if reviews from the microblog are very large [46, 47]. Recommendation frameworks were presented as agents of the second class, being characterized as frameworks that “… enable individuals to settle on decisions dependent on the conclusions of other individuals.” [48]. Early data-sharing frameworks had a place with the primary class and depended on text-based classification or separation, which works by choosing important things as per many literary catchphrases [49]. Recommender frameworks propose “things important to clients dependent on their unequivocal and verifiable inclinations, the inclinations of different clients, and client and thing traits.” [50]. The recommendation system is finding the right product according to the taste of the customer by filtering the fact through the likeness value [51]. Suggestions utilize the assessments of a community of clients to help people in that community all the more adequately distinguish the content of enthusiasm from a possibly overpowering set of decisions [52]. Recommendation by demographics which groups the users as per the traits of their personnel file, besides, creates proposals dependent on classes of the statistic. A premature precedent is a generalization-based Grundy system, which has been made to bolster book searching in a library [53]. The recommendation is reliant on the computation of utility of each item for a user' utility capacity (http://www.eqo.info). Recommendation by knowledge proposes things dependent on legitimate inductions about a user's inclinations. A learning portrayal or a rule about how a thing meets a specific client requirement is important (http://www.findme.com.ph). By applying preference-based collaborative filtering, a recommender system intend to foresee majority of estimation of likeness, where a few users may provide inconspicuous views as well [54]. There are two types of architecture for the recommendation systems: One is centralized and situated at a specific location [55]. Another one is geographically distributed and situated at different locations [56]. There are three types of recommendation modes by which the system will be initiated: The first one is the push mode in which suggestions are pushed to the user while he is not associating with the system by email [48]. The second one is the pull mode in which suggestions are generated but are displayed to the user just when he permits or unequivocally asks for it [57]. Push and pull modes are the active mode in which the recommender is initiated. The third one is the passive mode in which suggestions are generated as a feature of the customary framework activity, for instance, an item suggestion with reference to a user's preference [58]. A user's preference of items can be determined by using the linear adaptive function multiattribute utility theory (MAUT) [59]. Cosine similarity determined by cosine vector comparability is one of the well-known measurements of insight since it notionally considers just the edge of two vectors without the size. The collaboration between the search item and the other item that is rated by users can be measured by the angle of their vectors; if the angle is 90°, then the value of cosine similarity is zero, which means the item is irrelevant. If the angle between cosine vectors is nearly about zero, then the value of cosine similarity is one, which means the product is relevant (https://en.wikipedia.org/wiki/Cosine_similarity) [60]. There are three major classes of collaborative filtering: (1) collaborative filtering (CF) in which users and items' profile data are required to make a decision for recommendation [61], (2) content-based filtering on the description of the content of items and user preference information (explicate or implicate) for recommendation [62], and (3) combining various filtering techniques to handle scalability, sparsity, and cold start problem and other big data issues of the recommendation system to get better outcomes [63].

## 3. Multivariate Movie Recommendation Model

**Figure 1:**
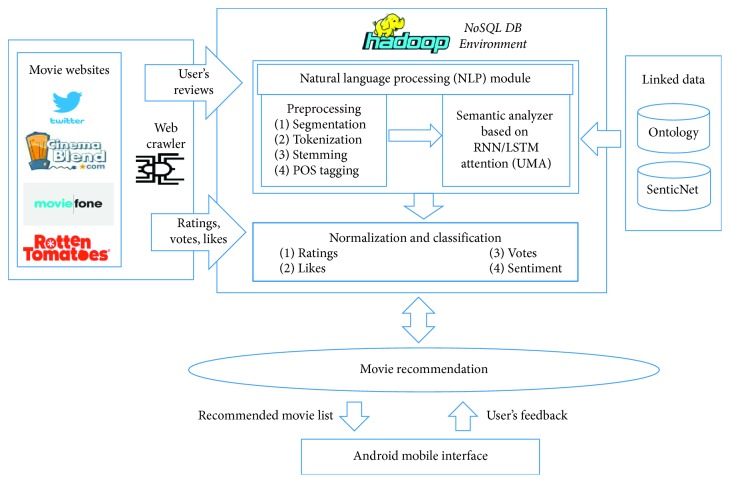
Architecture of the multivariate movie recommendation system.

The multivariate approach is (see Figure 1) based on three modules: mobile app, multivariate recommender, and web scraper. Users can get the recommendation services through a mobile application. The mobile app module provides the information such as the user's query, profile, and history to the recommender module. The recommendation is made for both registered and unregistered users of the mobile app. The recommendation module is based on the deep learning NLP module and computation module. The NLP module preprocesses the fetched qualitative data (user's reviews) of microblogs using a tokenizer, stemmer, and POStagger and then semantically analyzes the reviews and extracts the semantic emotions about movies. Semantic parser work is based on the deep machine learning algorithm recurrent neural network (RNN/LSTM attention) with user movie attention (UMA). Semantic emotion is classified into five major classes: (i) Highly Favorable, (ii) Favorable, (iii) Averagely Favorable, (iv) Unfavorable, and (v) Highly Unfavorable, on the bases of their relative semantic scores. While the computation module normalized the quantitative data (Twitter likes, votes, and ratings), normalized scores and semantic emotional scores were evaluated to generate the recommended movie list. The recommended movie list consists of five medals and their popularity such as Platinum: “Highly Popular,” Gold: “Popular,” Silver: “Averagely Popular,” Bronze: “Unpopular,” and Copper: “Highly Unpopular.” The recommended movie list is generated according to users' taste and preference. A web scraper fetched data (reviews, Twitter likes, votes, and ratings) from external data source sites (CinemaBlend, Moviefone, Rotten Tomatoes, and Twitter) and stored them in the NoSQL database for computation. Users' feedback about a movie and app is useful for generating the recommended list and evaluation of system reliability.

### 3.1. NLP Module

NLP has the capability to understand natural language. Users share their opinions and reviews from the microblog that help in making a decision. Positivity, negativity, and neutrality are extracted by opinion mining, whereas emotions are extracted by semantic analysis. In our work, the NLP module determines the semantic emotion of the movie's reviews by the LSTM-attention machine learning algorithm. This semantics is one of the parameters in multivariates used to make a recommendation. This methodology for semantics is depicted as follows:

- The module fetches the reviews from microblogs related to movies such as CinemaBlend, Moviefone, and Rotten Tomatoes
- The module preprocesses the microblog text or reviews using a sentence splitter, tokenizer, and stemmer/lemmatizer
- The module determines the sense of the word to strength the sentiment using SenticNet
- Semantic parsing based on attention is done to construct a parse tree to identify the syntactic tree as the emotion of the sentence
- RNN/LSTM-user movie attention (UMA) machine learning algorithm is used to classify the reviews

### 3.2. Preprocessing

It is estimated that more than 80% of data are unstructured and not in an organized manner. Preprocessing of text is cleaning or normalization of text/reviews. Stemming or lemmatization and tokenization are done to reduce the sparsity and shrink the feature space. Semantic analysis has to face some challenges such as short text, misspelling, grammatical mistake, slang, unusual terms, tags, white spaces, noise, and emoji. Text is a sequence of words, while word is a meaningful sequence of characters. However, the question is how to find out the boundaries of words. Words are identified by spaces or punctuation in English. However, a compound word is a set of words which have no spaces in German, for example, (“childhood memories description of an unforgettable event”) ⟶ (“Kindheitserinnerungen Beschreibung eines unvergesslichen Ereignisses”), while there are no spaces at all in Japanese like this (“childhoodmemoriesdescriptionofanunforgettableevent”).

#### 3.2.1. Tokenization

The process of splitting the text stream into units is called tokenization. Units refer to tokens. For example, “This movie is so riddled” is a character string which is tokenized as [This] [movie] [is] [so] [riddled]. Splitting the input sequence into tokens has some problems. Splitting by white space has a problem that different tokens are tokenized into similar words, while the same words may have similar meanings (https://NLTK.Tokenize.WhiteSpaceTokenizer). Splitting by punctuation in which some punctuation are not meaningful is like “An apostrophe problem” (https://NLTK.Tokenize.WordPunctTokenizer). Splitting comes up with the set of rules that generate a more meaning full result (https://NLTK.Tokenize.TreeBankWordTokenizer).

#### 3.2.2. Stemming (Lemmatization)

The stemmer stemmed the words like the Porter stemmer, which stemmed the English words “looked” as “look” with a morphological production rule, for example, [(“SSES ⟶ SS”): (“Caresses ⟶ caress”)], [(“IES ⟶ I”): (“Ponies ⟶ Poni”)], [(“SS ⟶ SS”): (“Caress ⟶ Caress”)], and [(“S ⟶ S”): (“Cats ⟶ Cat”)], but due to stemming of nonwords, the same plural word can be stemmed to singular and irregular forms. These are produced like (Wolves ⟶ wolv), (Feet ⟶ Feet). The WordNet database is looked up for lemmas to solve this type of problem. It solves some specific problems but not all, like (Wolves ⟶ wolf) and (Feet ⟶ Foot) (https://NLTK.Stem.WordNetlemmatizer).

#### 3.2.3. POS-Tag Generation

POS tags are determined for all the tokens by Treebank POStagger. Treebank Project 1 represents 36 POS tags (http://www.ling.upenn.edu/courses/Fall_2003/ling001/penn_treebank_pos.html). For example, the POStag of string “Unwatchable I made it through 20 minutes I think” is [Unwatchable/VB] [I/PRP] [made/VBD] [it/PRP] [through/IN] [20/CD] [minutes/NNS] [I/PRP] [think/VBP].

#### 3.2.4. Word Sense Disambiguation (WSD)

WSD is the issue of deciding the “sense” of a word. A lexicon controls a word and its conceivable faculties. Bar-Hillel, 1960, presented the example [“Little John was looking for his toy box. Finally, he found it. The box was in the pen. John was very happy.”]. In the previous string, a word “pen” has different senses according to WordNet. “Pen” word defines an “ink flow from a point to write”; here, pen is defined as an “arena of cattle” and as a “bird's family.” In the assessment of the movie's reviews, SenticNet is utilized to indicate their degrees of polarity, antagonism, and impartiality. The SenticNet score of the terms and its recurrence are determined to get the general supposition of the reviews (https://sentic.net) [64].

### 3.3. Parsing

In NLP, parsing is the process of determining the structure of a sentence by analyzing its essential words based on an underlying syntax. The Stanford parser is used to construct the parse tree that determines the syntactic structure relative to grammar (language). Parsing can refer to various things. Shallow parsing or chunking is the process of grouping the words into noun phrases (NP). Stuff can also be grouped into VP (verb phrases) and PP (prepositional phrases) using grammar like (S ⟶ NP│VP), (NP ⟶ DetNoun), (NP ⟶ ProperNoun), and (VP ⟶ Verb│NP). In contrast, dependency parsing determines the dependencies between the words and their type. For example, spaCy + displaCy for parsing and rendering is used to produce a more semantic result.

#### 3.3.1. RNN/LSTM

**Figure 2:**
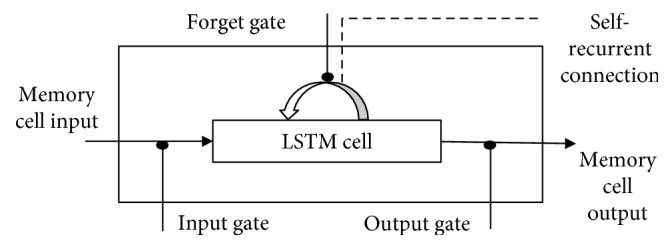
LSTM cell architecture.

Neural networks are represented by RNN/LSTM cells [65]. Typically, in Birdseye, RNN/LSTM is a chain of several copies of the same static network, as shown in Figure 2. From input, the sequence of copies of networks is working in a single timestep. In addition, networks are linked with each other via their hidden states *h*. So we can say that every copy network has its own inputs as the copy network is unfolded or unrolled. Let the sequence be represented as *x*_1_, *x*_2_, *x*_3_ … *x*_*n*_ and each timestep be represented as *x*_*t*_ ∈ *x*_1_ … *x*_*n*_. At timestep *t*, *h*_*t*_ is a hidden layer and *f* is used to calculate the hidden state: *h*_*t*_ = *f*(*h*_*t*−1_, *x*_*t*_). A word is represented by a timestep in the long sequence. For example, the given string is represented as a sequence in the mathematical form: “it is a good movie” ⟶ [“it,” “is,” “a,” “good,” “movie”]. And the timestep (*t* = 0, 1, 2,….) for the string “it” is represented as *x*_0_, “is” as *x*_1_, “a” as *x*_2_, “good” as *x*_3_, and “movie” as *x*_4_. If *t* = 1, then *x*_*t*_ = “is” ⟶ “current timestep to event” and *x*_*t*−1_ = “it” ⟶ “previous time stamp to event”:

(1) $$\begin{array}{c} X=\left(\begin{array}{c} h_{t-1}\\ x_{t} \end{array}\right),\\ \text{input gate at time }t:\;i_{t}=\sigma \left(W_{i}\cdot X+b_{i}\right),\\ \text{forget gate at time }t:\;f_{t}=\sigma \left(W_{f}\cdot X+b_{f}\right),\\ \text{candidate state at time }t:\;{\tilde{C}}_{t}=\tanh\left(W_{c}\cdot X+b_{c}\right),\\ \text{final memory cell}:\;C_{t}=f_{t}\;*\;c_{t-1}+i_{t}\;*\;{\tilde{C}}_{t},\\ \text{output gate}:\;o_{t}=\sigma \left(W_{o}\cdot X+b_{o}\right),\\ h_{t}=o_{t}\;*\text{ tanh}\left(C_{t}\right). \end{array}$$

At the *i*_*t*_*s* input gate, the decision on which information should be remembered or rid of is made by the sigmoid function *σ*. It produces a 0 or 1 value: 0 means forget, while 1 means remember in the cell state. Sigmoid function at the input gate takes a decision on which value should be updated, and the new candidate value information is represented by tanh function ${\tilde{C}}_{t}$. Output gate sigmoid function decides which part of information should be produced, and then tanh function produces the value between 1 and −1.

The sequential semantic information is preserved in the recurrent neural network's hidden states. In the hidden state (*h*_*t*_), the semantic information of the input sequence is preserved. When a new input is experienced and again delivered to be the subsequent input, then semantic information is altered. Passing the information from one to another network helping to find out the correlation among the words from the sequence is represented as a long-term dependency.

#### 3.3.2. LSTM-Based Sequence Labeling

Predicates from a given input sequence are marked, and the label arguments corresponding to every predicate are identified. For example, in the given sentence “I watched the movie,” the predicate (watched) is marked, and labels corresponding to the predicate are “I,” “the,” and “movie” as an agent, null, and theme, respectively. Multiple predicates may present in a sentence, and different labels may be marked to the same word for every predicate. Concatenating pretrained ones (Word2vec) generates vectors of every word. The 1-bit flag represents the predicate in the specific training unit to confirm that the network deals with every predicate separately and serves it into the LSTM layer to the word context. With the predicate, any one word is labeled to take the dot product of its hidden state. A softmax function is applied over it. The probability of a sentence is calculated as follows:

(2) $$\begin{array}{c} P\left(X\right)=\overset{n}{\underset{k=1}{\prod }}P\left(x_{i}\;|\;x_{1},x_{2},x_{3}\ldots \;x_{t-1}\right),\\ X_{l,r}=\text{ReLU}\left(x_{l}\cdot x_{r}\right). \end{array}$$

Here, the role label *r* is calculated by the weight matrix parameter using ReLU function and predicate lemma and the role depicted by taking the dot product of vectors to embedding.

#### 3.3.3. Neural Sentiment Classification (NSC)

**Figure 3:**
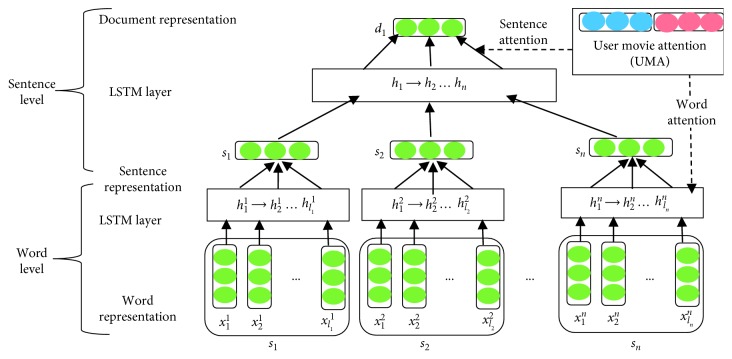
LSTM-attention (UMA) architecture.

Document-level sentiment classification is measured by neural sentiment classification (NSC) based on hierarchical LSTM attention with user movie attention (UMA) (see Figure 3) that is represented by the user's global information and movie features [28]. Let a review *d* ∈ *D* with sentences, each sentence (*s*_1_, *s*_2_,   …,  *s*_*n*_) of a particular review *s*_*i*_ ∈ *d*, a user *u* ∈ *U*, and a movie *m* ∈ *M* review corpus (users and their movie set). Moreover, *l*_*i*_ is the length of the  *i*-th sentence, while *s*_*i*_ consists of *l*_*i*_ words as *x*_1_^*i*^, *x*_2_^*i*^,…, *x*_*l*_*i*__^*i*^. Predicting the semantic rating of documents is done according to their text information. Firstly, in word-level low-dimensional semantic space, each word *x*_*j*_^*i*^ is mapped to its embedding *x*_*j*_^*i*^ ∈ *ℝ*^*d*^ in a sentence. Every step has a given input word *x*_*j*_^*i*^, the current cell state *c*_*j*_^*i*^, and the hidden state *h*_*j*_^*i*^ that may be updated with the preceding cell state *c*_*j*−1_^*i*^. Then, the hidden state *h*_*j*−1_^*i*^ is represented. The document representation architecture is presented as follows:

(3) $$\begin{array}{c} \left[\begin{array}{c} i_{j}^{i}\\ f_{j}^{i}\\ o_{j}^{i} \end{array}\right]=\left[\begin{array}{c} \sigma \\ \sigma \\ \sigma \end{array}\right]\left(W\cdot \left[h_{j-1}^{i},x_{j}^{i}\right]+b\right),\\ {\hat{c}}_{j}^{i}=\tanh\left(W\cdot \left[h_{j-1}^{i},x_{j}^{i}\right]+b\right),\\ c_{j}^{i}=f_{j}^{i}\;⊙\;c_{j}^{i}+i_{j}^{i}\;⊙\;{\hat{c}}_{j}^{i},\\ h_{j}^{i}=o_{j}^{i}\;⊙\;\tanh\left(c_{j}^{i}\right). \end{array}$$

Sigmoid activation function and gate activation functions are represented as *σ* and *i*,  *f*, and *o*, respectively, while elementwise multiplication is represented as ⊙. Training parameters needed for training are represented as  *x* and  *b*. The feed hidden states [*h*_1_^*i*^, *h*_2_^*i*^,…, *h*_*l*_*i*__^*i*^] are represented to a mediocre pooling layer to acquire the representation of the *s*_*i*_ sentence. Sentences are embedded at the sentence level (*s*_1_, *s*_2_,…, *s*_*n*_) into the LSTM; after that, document representation *d* is acquired via a mediocre pooling layer in a similar way as follows:

(4) $$\begin{array}{c} \left[\begin{array}{c} i_{i}\\ f_{i}\\ o_{i} \end{array}\right]=\left[\begin{array}{c} \sigma \\ \sigma \\ \sigma \end{array}\right]\left(W\cdot \left[h_{i-1},s_{i}\right]+b\right),\\ {\tilde{C}}_{i}=\tanh\left(W\cdot \left[h_{i-1},s_{i}\right]+b\right),\\ C_{i}=f_{i}\;⊙\;c_{i-1}+i_{i}\;⊙\;{\tilde{C}}_{i},\\ h_{i}=o_{i}\;⊙\text{ tanh}\left(\;C_{i}\right). \end{array}$$

Here, training parameters needed for training are represented as *s* and  *b*. The feed hidden states [*h*_1_, *h*_2_,…, *h*_*n*_] are represented to a mediocre pooling layer to acquire the *d*_*i*_ document representation.

#### 3.3.4. User Movie Attention (UMA)

At various levels, a necessary component is extracted by using user movie attention (UMA) for sentiment classification. UMA is applied at the word level to construct a sentence and sentence level to generate a document. Obviously, sentence meaning may not be represented by all words for several users and movies. In spite of feeding hidden states at the word level to an average pooling layer, user movie attention (UMA) is used to extract user/movie relative words, which are essential to sentence meaning. Informative words are aggregated to produce the representation of the sentence. Formally, weighted hidden states generate the enhanced sentence as follows:

(5) $$\begin{array}{c} s_{i}=\overset{l_{i}}{\underset{j=1}{\sum }}a_{j}^{i}h_{j}^{i},\\ d_{i}=\overset{n}{\underset{i=1}{\sum }}a_{i}h_{i}. \end{array}$$

Importance of the *j*th word is measured by *a*_*j*_^*i*^ for the current user and movie. Each user u and movie m are embedded continuous and real-valued vectors *u* *ε* *ℝ*^*d*_u_^ and *m* *ε* *ℝ*^*d*_m_^, while user and movie embedding is represented as *d*_u_ and *d*_m_ dimensions, respectively. Moreover, for every hidden state, the attention weight *a*_*j*_^*i*^ is presented as follows:

(6) $$\begin{array}{c} a_{j}^{i}=\frac{\exp\left(e\left(h_{j}^{i},u,m\;\right)\right)}{\sum_{k=1}^{l_{i}}\exp\left(e\left(h_{k}^{i},u,m\;\right)\right)}. \end{array}$$

For the sentence level,

(7) $$\begin{array}{c} a_{i}=\frac{\exp\left(e\left(h_{i},u,m\;\right)\right)}{\sum_{i=1}^{n}\exp\left(e\left(h_{i},u,m\right)\right)}. \end{array}$$

Importance of words for sentence representation as well as document representation is presented by *e* score function as follows:

(8) $$\begin{array}{c} e\left(h_{j}^{i},u,m\right)=v^{\text{T}}\;\tanh\left(W_{h}h_{ij}+W_{u}u+W_{m}m+b\right). \end{array}$$

For the sentence level,

(9) $$\begin{array}{c} e\left(h_{i},u,m\right)=v^{\text{T}}\;\tanh\left(W_{h}h_{i}+W_{u}u+W_{m}m+b\right), \end{array}$$

where *v* is a weight vector and *v*^T^ represents its transpose, while *W*_*h*_, *W*_*u*_, and *W*_*m*_ are weight matrices. Meaning of every document varies for different users and movies by the sentence, which provides the hints. So in the sentence level, usage of attention *a* with the *u* user and *m* movie vector at the word level to select informative sentences to generate document representation *d* is presented as follows:

(10) $$\begin{array}{c} d=\overset{n}{\underset{i=1}{\sum }}\beta_{i}h_{i}. \end{array}$$

In the sentence level, the *β*_*i*_ weight of the *h*_*i*_ hidden state is measured similar to word attention. The higher level representation of document *d* is generated by hierarchical extraction from words and sentences in the document. So, for sentiment classification of the document, it is used as features. tanh activation function is used at the nonlinear layer for current document representation in the target space of *C* classes:

(11) $$\begin{array}{c} \hat{d}=\tanh\left(W_{c}d+b_{c}\right). \end{array}$$

tanh activation function is used at an absolute layer to get sentiment distribution of the document:

(12) $$\begin{array}{c} d_{c}=\frac{\exp\left({\hat{d}}_{c}\right)}{\sum_{k=1}^{C}\text{exp}\left({\hat{d}}_{k}\right)}. \end{array}$$

Sentiment classes and prediction probability of sentiment class *C* are represented as *C* and *p*_*c*_, respectively. During the training, loss function for optimization is measured by error cross-entropy between the distribution of Gold sentiment and distribution of our model sentiment as follows:

(13) $$\begin{array}{c} L=\underset{d\varepsilon D}{\sum }\overset{C}{\underset{c=1}{\sum }}p_{c}^{g}\left(d\right)\cdot \log\left(p_{c}\left(d\right)\right). \end{array}$$

Here, Gold probability of sentiment class *C* and training document are represented as *p*_*c*_^*g*^ and *d*, respectively, while reality-based truth is one and others are zero.

Some nomenclatures used in our mathematical model are presented in Table 1.

Table 2 presents the emotion class.

### 3.4. Computation and Classification

Sentiment analysis determines the emotions of reviews. Firstly, the aggregated sentiment score of each document from each site for the *j*-th movie is computed. Then, the qualitative score and then aggregated quality scores for Twitter likes are computed to get the final score for the recommendation of movies and generate the popularity class relative to the final score:

(14) $$\begin{array}{c} C^{j,1}=\underset{k=1}{\overset{n}{\underset{i=0}{\sum }}}dc_{i}^{j,k},\\ C^{j,2}=\underset{k=2}{\overset{n}{\underset{i=0}{\sum }}}dc_{i}^{j,k},\\ C^{j,3}=\underset{k=3}{\overset{n}{\underset{i=0}{\sum }}}dc_{i}^{j,k},\\ q^{j,1}=\left[\left(R^{j,1}\right)+\left(V^{j,1}\right)+\left(C^{j,1}\right)\right],\\ q^{j,2}=\left[\left(R^{j,2}\right)+\left(V^{j,2}\right)+\left(C^{j,2}\right)\right],\\ q^{j,3}=\left[\left(R^{j,3}\right)+\left(V^{j,3}\right)+\left(C^{j,3}\right)\right],\\ Q^{j}=\left[\left(q^{j,1}\right)+\left(q^{j,2}\right)+\left(q^{j,3}\right)\right],\\ Q^{j}=\overset{3}{\underset{k=1}{\sum }}q^{j,k},\\ j‐\text{th movie final multivariate emotional score }E=\log\left(\gamma \left[\left(Q^{j}\right)+\left(\text{TL}\right)\right]\right), \end{array}$$

where *γ*=0.5, and

(15) $$\begin{array}{c} j‐\text{th movie popularity score }P=\left[\frac{f_{m_{j}}-\min\left(f_{m_{j}}\right)}{\max\left(f_{m_{j}}\right)-\;\min\left(f_{m_{j}}\right)}\;*\;10\right]. \end{array}$$

This mathematical formulation is used to determine the final popularity score using the multivariate model. The emotional value is stretched to 10 scales, by which the popularity status is determined. Every movie is labeled with a medal according to the popularity score, and the algorithm that identifies the medal by using fuzzy logic on behalf of the popularity score to find the popularity of the movies is depicted as follows:

1. IF multivariate value ≥08, THEN: Platinum: “Highly Popular”
2. ELSE IF multivariate value ≥06, THEN: Gold: “Popular”
3. ELSE IF multivariate value ≥04, THEN: Silver: “Average Popular”
4. ELSE IF multivariate value ≥02, THEN: Bronze: “Unpopular”
5. ELSE Copper: “Highly Unpopular”

The ranges of the popularity scores and their respective medals and degree of popularity are given in Table 3.

The category represented by a movie genre to classify the movie according to its features, movie recommendation services suggests top 10 popular movies with their category according to the user request and profile history.

## 4. Multivariate Movie Recommendation System Implementation

### 4.1. System Component Interaction

**Figure 4:**
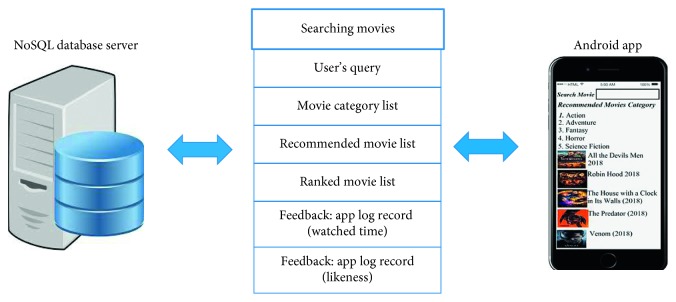
Interaction of components of multivariate movie recommendation.

User android application is front end of the system (see Figure 4) by which users can get the web services from the system, and back end is the movie recommendation system in the NoSQL environment with Apache Mahout and Hadoop, which provide the web services to the users as well as a web scraper by which the system fetched the data. Web scraper fetched the data from the external data source on the bases of matching lexicons of the query and movie content.

### 4.2. NoSQL Environment Implementation

#### 4.2.1. Hadoop Architecture

It is a framework with four fundamental components: (1) HDFS splits the file into many small files and stores them on three servers for fault tolerance constraints as replicas in a distributed file system manner. (2) Map Reduce programming standard is for handling and manipulating big data. (3) Common/Core holds the reference library and services to backing up Hadoop. (4) YARN performs management, computation, and scheduling of resources and tasks.

#### 4.2.2. Apache Mahout

Implementation of collaborative filtering, clustering, and classification is done by Apache Mahout. In the NoSQL environment, Apache Mahout interfaces implement the Hadoop framework and evaluate the performance similarities and neighborhood measures. A multivariate web scraper is implemented and big data are generated.

### 4.3. Web Scraper

Our web scraper is a scripting program, which surfs the W3, fetches data from different movie websites to extracts the reviews, votes, ratings, and Twitter likes, and stores them in the repository. In addition, it manages and handles scrape data in a NoSQL environment using Hadoop and Apache Mahout. The web scraper (web bot) receives the URLs and matches them with keywords (Meta tags) of the web page. If the keywords are matched, then the web pages are downloaded; otherwise, the irrelevant pages are discarded.

### 4.4. NLP Tools

Stanford CoreNLP technology tools are used to process the natural language like English. They give the words, relative parts of speech, and identification of sentiments. The Stanford CoreNLP framework is the integration of many of Stanford's NLP tools, like POStagger, parser, sentiment analyzer, named “entity recognizer,” and pattern learning and information extracting tools from https://stanfordnlp.github.io/CoreNLP.

### 4.5. Mobile Application Usage

#### 4.5.1. Unregistered Users

Unregistered users can request the movie by the search query to our recommendation system, and the system will respond to that query by content filtering to extract the features or content of a movie from the query. Collaboration is done between the user request and system-generated movies. The system provides the watched window to unregistered users for watching the recommended movies. Unregistered users give feedback by their likeness, and the system uses the feedback for accurate measurement.

#### 4.5.2. Registered Users

If unregistered users sign up, then they can sign in and maintain their profile or history. For registered users, collaborations may be done on the bases of both the query and the history. Registered users may provide feedback to the system by their likeness, and the system uses this feedback for collaborative filtering between liked movies or their movie history and system movies for recommendations of the movie of their choices as well as accurate measurement of the multivariate movie recommendation system. The application also provides a watch window for registered users to watch the recommended movie.

### 4.6. Cold Start Problem Handling

Collaborative filtering (CF) is done for movie recommendation for registered and unregistered users; but in two cases, the problem may occur:

- Case 1: if the registered users request the movie, the system collaborates the requested movie with the system movie and recommends the movies on behalf of user history. Here, one problem arises: if the newly registered users request the movies, then the system recommends the movies according to movies mostly liked by others to solve the cold start problem of newly registered users.
- Case 2: if a new movie arrives for registered or unregistered request, then the system recommends the movies according to the collaboration of new movie trailers, which were mostly liked to solve the cold start problem of newly released movies.

### 4.7. Similarity Measurement

**Figure 5:**
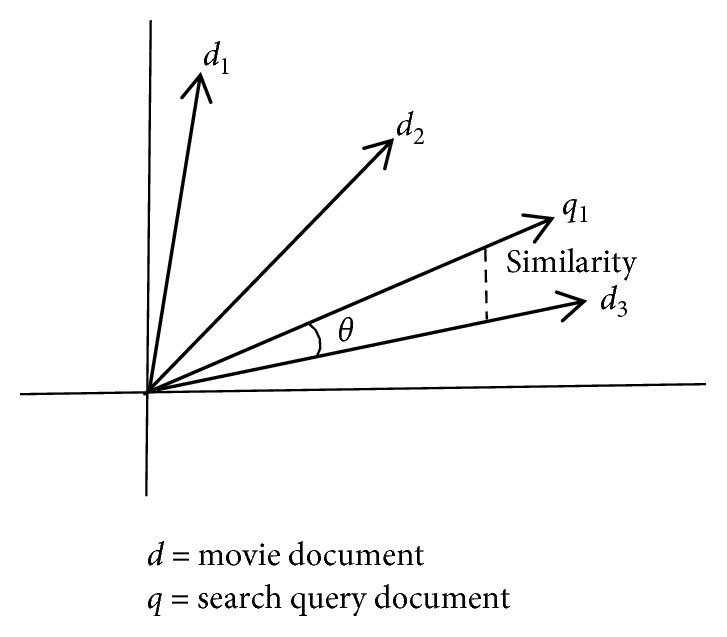
Cosine similarity angle. *Note.* If the angle between vectors is zero degrees, then the cosine similarity value is 1, which means movies are similar or relevant, and if the angle is 90 degrees or above, then the similarity value is zero, which means the movie is irrelevant.

We use cosine similarity in which there are two vectors for measuring the angle value for similarity manipulation. A smaller angle degree is directly proportional to larger similarity, and vice versa, as shown in Figure 5. It is also known as vector-based similarity. Movie document and search query document correlation is computed where *q* is the search query document and *d* is the movie document. The similarity can be calculated by the following equation:

(16) $$\begin{array}{c} \vec{q}\cdot \vec{d}=\left\|\vec{q}\right\|\cdot \left\|\vec{d}\right\|\cdot \cos\;\theta ,\\ \text{sim}\left(q\cdot d\right)=\cos\;\theta =\frac{\vec{q}\cdot \vec{d}}{\left\|\vec{q}\right\|\cdot \left\|\vec{d}\right\|}. \end{array}$$

## 5. Experiments and Results

The procedure followed by data preprocessing is NLP procedures applied for sentiment analysis on fetched data, and then the sentiment score is computed by using SenticNet and obtained results are presented as follows.

Table 4 presents the identification of movies and categories.

Table 5 presents the identification of sites, movies, users, and reviews.

Table 6 presents the movie review from the movie website CinemaBlend.

Movie reviews from movie websites Moviefone and Rotten Tomatoes were also fetched, and semantic scores and emotions were computed.

Table 7 presents the movie review tokenization and tagging from movie websites CinemaBlend, Moviefone, and Rotten Tomatoes.

The parsing of movie reviews' tokens taken from CinemaBlend, Moviefone, and Rotten Tomatoes was performed using the Stanford parser. Here, Table 8 presents the sentiment score of movie reviews from CinemaBlend, Moviefone, and Rotten Tomatoes.

Table 9 presents the normalized Twitter likes of movies from Twitter.

Table 10 presents the normalized rating score of movies from CinemaBlend, Moviefone, and Rotten Tomatoes.

Table 11 presents the normalized vote score of movies from CinemaBlend, Moviefone, and Rotten Tomatoes.

Table 12 presents the final score, movie category, medal rank, and genres of movies from CinemaBlend, Moviefone, and Rotten Tomatoes.

**Figure 6:**
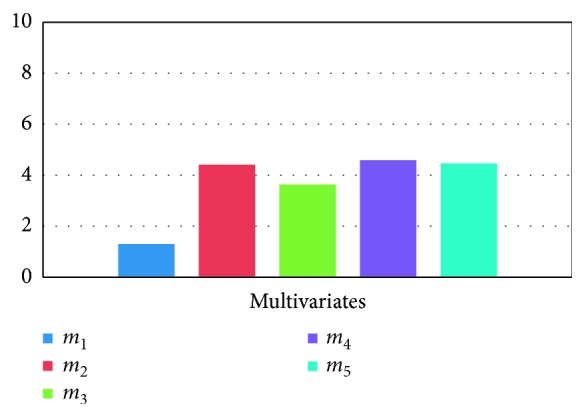
Multivariate movie ranked recommendation.

Figure 6 presents the multivariate movie ranked recommendation of movies from CinemaBlend, Moviefone, and Rotten Tomatoes.

**Figure 7:**
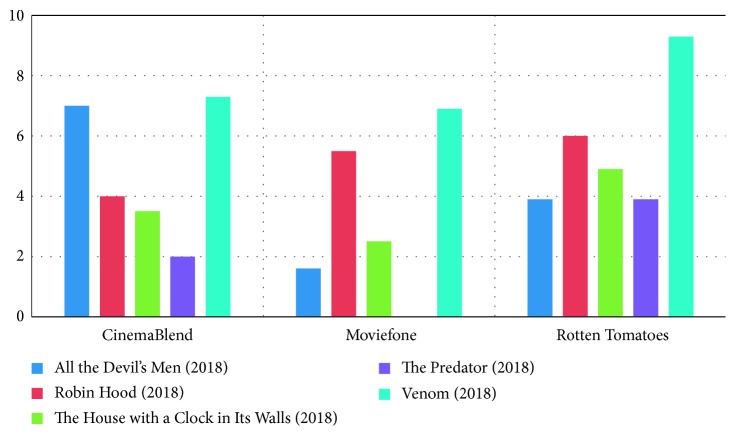
Rating difference.

Figure 7 presents differences in the rating of the movie from CinemaBlend, Moviefone, and Rotten Tomatoes.

**Figure 8:**
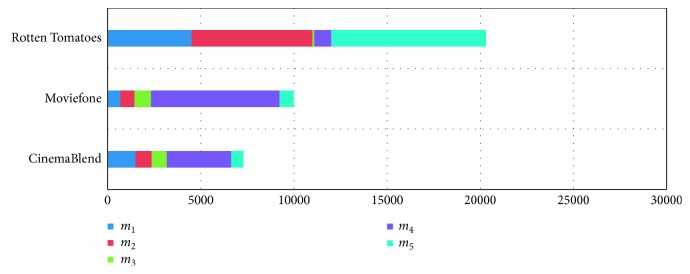
Vote difference.

Figure 8 presents differences in the votes of the movie from CinemaBlend, Moviefone, and Rotten Tomatoes.

**Figure 9:**
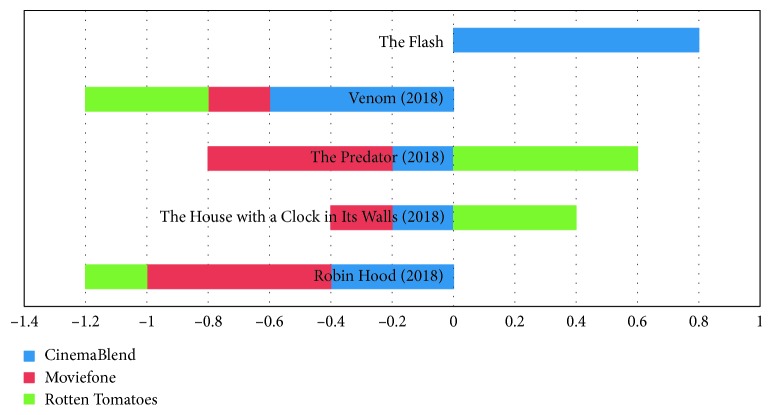
Differences in sentiment scores.

Figure 9 presents differences in the sentiment of the movie from CinemaBlend, Moviefone, and Rotten Tomatoes.

## 6. Evaluation and Discussion

We evaluate the sentiment classification models as well as recommendation models as follows.

### 6.1. Sentiment Classification Model Evaluation

For sentiment, classification models are evaluated by accuracy and RMSE, which measure the overall performance of the sentiment classification model and divergence between predicted and truth ground sentiment classes, respectively. We compare the several base sentiment classification methods using three datasets imdb, yulp13, and yulp14, which contain reviews about movies using Stanford CoreNLP. Majority of the baseline sentiment classification models refer to categorization of document sentiments in the training set by an *SVM* classifier with unigram, bigram, and trigram. Text feature extraction including character *n*-gram and -word is done by the SVM classifier. Use of leniency feature is extracted by UPF [66]. Document representation is obtained by AvgWordvec, which nourished into SVM. Feature generation is by SSWE (sentiment-specific word embedding) [67]. Sentence representation is by the RNTN (recursive neural tensor network) [68]. Document classification is by paragraph vector: distributed memory model (PVDM) [69], topic modeling, and collaborative filtering JMARS (https://jmars.asu.edu/). Sentiment classification is by vector representation and text preference matrix for the user product neural network (UPNN) [70].

Table 13 presents the comparison between different sentiment classification models using and without using users/product/movies information.

In our approach, the core implementation is neural sentiment classification (NSC) using local user and movie information [71], which provides the significant result, as shown in Table 13, which represents the significant 4% improvement/difference with all the baseline methods, which use the local textual information about users and movies. While using global information about users and movies, the UPNN gains 3% improvement, and our approach NSC-UMA achieves 9% improvement. Our approach uses the vector for embedding the user and movie information, which is suitable for larger datasets, while the UPNN uses the matrix and vector simultaneously. NSC-UMA is considerable for capturing the information from each semantic layer. Therefore, our model incorporates using user movie global information in an efficient and effective way.

Word-level attention and sentence-level attention are considerable to outperform to reflect the semantic information of user and movie characteristics at multiple levels, which leads to introduction of the user movie attention (UMA) in sentiment classification. Furthermore, perceptions of user taste or preferences are more understandable than movie attributes, so both user and movie information is essential to pay attention in the document for semantic information which impacts movie ranking for recommendation.

### 6.2. Comparative Analysis of Recommendation Models

Table 14 presents the comparison between different models. Major differences which show our work as a novel approach are that the first one is LSTM-UMA for sentiment classification, the second one is the NoSQL distributed environment to deal with the big data issues, the third one is the multivariate (qualitative and quantitative) score fetched by a web bot from three different reliable external data sources, and the fourth one is app features (movie category and popularity).

In [15] which only uses the implicate and explicate ratings, no user and production attention are used by LSTM, adoptive deep learning is not used to determine the preference and taste of users about a movie from microblogs, so we can say that the authors did not use the qualitative data. It does not declare how data are fetched nor categorized with their popularity. In [15, 72–74], multivariates are not used, and the study [73] is just based on microblogs, while the study [74] uses movie feature ratings.

### 6.3. Results of the Experiments

True positive (recommended interesting movie) predicted by a search divided by actual movies (total movies) is called precision:

(17) $$\begin{array}{c} \text{precision}=\frac{\text{TP}}{\text{TP}+\text{FP}}. \end{array}$$

True positive (recommended interesting movie to users) predicted by a search divided by predicted movies (totally recommended movies) is called recall:

(18) $$\begin{array}{c} \text{recall}=\frac{\text{TP}}{\text{TP}+\text{FP}}. \end{array}$$

**Figure 10:**
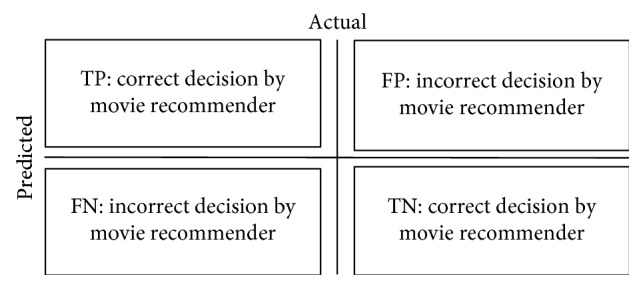
Different decisions made by the movie recommender. TP: the recommended movie is interesting. TN: the recommended movie is uninteresting. FP: the recommended movie is actually interesting. FN: the recommended movie is actually uninteresting.

Figure 10 presents different decisions made by the movie recommender.

If the recommender grows in precision, then recall is declined:

(19) $$\begin{array}{c} F\text{ score}=2\cdot \frac{\text{precision}\cdot \text{recall}}{\text{precision}+\text{recall}}. \end{array}$$

Table 15 presents the comparisons of the parameters of the multivariate recommendation system and other models.

Table 16 presents the evaluation of the multivariate recommender system with other parameters and other works.

The results of *F* score in our system are compared with other predicting parameters as well as with other recommendation frameworks. The accuracy of the multivariate system is nearly about 98.70%. The true positive rate of the multivariate system is 0.99106, which means the system recommended movies truly interested for users, and the false positive rate is 0.01814, which means the multivariate system did not recommend movies truly not interested for users.

**Figure 11:**
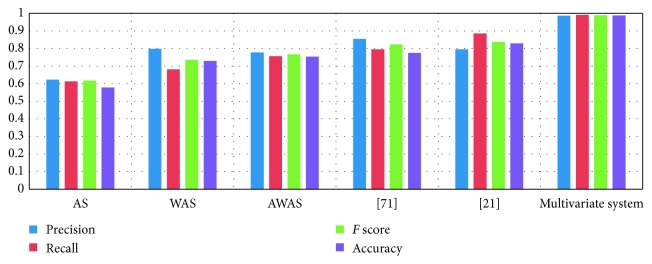
Differences in different decision parameters precision, recall, *F* score, and accuracy and recommendation models.

Figure 11 presents differences in different decision parameters (precision, recall, and accuracy) for recommendation of movies from movie sites CinemaBlend, Moviefone, and Rotten tomatoes and other recommendation models.

Figure 11 justifies the difference between our approach and other works [21, 74]. In [21], just finding the polarity of the term is not enough to evaluate the reviews for significant recommendation, and in [74], LSTM is used to determine the user and movie information, while we used NSC-UMA to evaluate the sentiment score of reviews for significant recommendation.

## 7. Conclusion

In many movie recommendation systems, suggestion and ranking are done on the bases of only likes or ratings. Furthermore, most of the systems extract data from only one or two sites. Semantics, ratings, or votes for the movie ranking are not reliable and insignificant as they could not provide better recommendation services and there is a huge gap between statistical information (ratings, votes, and likes) and reviews of movie websites; so they are not reliable using from one site as a few of the websites are producing the qualitative score showing high popularity of a movie and other ones are showing low popularity of the same movie. In study [21], deep learning is not used and only word frequency is used. Word frequency is no better way to evaluate the reviews semantically. It only produces the polarity of the term. Therefore, significant values and semantic information are required in an efficient way using the LSTM-attention learning algorithm for better semantic analysis, so semantic emotional value increases the significance of the recommendation system. Document semantic classification is improved by using the user movie attention at the word and sentence levels by the average pooling of word and sentence level to improve the semantic and emotion information about reviews or document. The reason for applying the attention at the word level is to improve the semantic information of the document as compared to only applying it at the sentence level. Big data issue is covered by adopting the NoSQL environment.

## 8. Future Work

This work may be enhanced by adding more parameters like session, playlist, users group, session, smiley, tag, context, the feature of movie and video content to improve the work.

## Figures and Tables

**Table 1:** Nomenclatures and description.

Nomenclature	Description
*d*	Document/review
*s*	Sentence
*x*	Word
*D*	Review corpus
*m*	Movie
*l*	Length of a sentence
*h*	Hidden state
*S*	Total movie sites
*b*	Biases
TL	Twitter likes
*t*	Timestep
*C* ^*j*,1^	*j*-th movie sentiment at site *S*_1_
*C* ^*j*,2^	*j*-th movie sentiment at site *S*_2_
*C* ^*j*,3^	*j*-th movie sentiment at site *S*_3_
*R* ^*j*,1^	*j*-th movie rating at site *S*_1_
*R* ^*j*,2^	*j*-th movie rating at site *S*_2_
*R* ^*j*,3^	*j*-th movie rating at site *S*_3_
*Q* ^*j*^	*j*-th movie total quantitative score
RecS	Final recommendation score
AWAS	Aggregated weighted average sentiment
Multivariate	Multivariate final score
*i*	Input gate
*o*	Output gate
*f*	Forget gate
*σ*	Activation function
*b*	Biases
*v*	Weight vector
*v* ^T^	Vector transpose
*t*	Timestep
⊙	Multiplication
*h*_*t*_	Hidden state at *t* timestep
*h*_*t*−1_	Hidden state at *t*−1 (previous) timestep
*W*	Weight matrix for input to hidden layers at *t* timestep
∅	tanh is an activation function
*x*_*j*_^*i*^	Input at timestep (*t*)
*V* ^*j*,1^	*j*-th movie votes at site *S*_1_
*V* ^*j*,2^	*j*-th movie votes at site *S*_2_
*V* ^*j*,3^	*j*-th movie votes at site *S*_3_
*L*	Loss
AS	Aggregated sentiment
WAS	Weighted average sentiment

**Table 2:** Emotion status.

Semantic score	Emotional class
0.5< and ≤1.00	Highly Favorable
0.00< and ≤0.5	Favorable
−0.5< and ≤0.00	Average Favorable
−1.00< and ≤−0.50	Unfavorable
≤−1.00	Highly Unfavourable

**Table 3:** Popularity scores and their respective medals and popularity status.

Popularity score	Medal rank	Status
0.8–1.0	Platinum	Highly Popular
0.6–0.79	Gold	Popular
0.4–0.59	Silver	Average Popular
0.2–0.39	Bronze	Unpopular
0.0–0.19	Copper	Highly Unpopular

**Table 4:** Movie ID and movie category ID.

Movie and category IDs		
Movie ID	Movie title	Movie category
*m*_1_	Robin Hood (2018)	Action (*c*_1_)
*m*_2_	The House with a Clock in Its Walls (2018)	Adventure (*c*_2_)
*m*_3_	The Predator (2018)	Fantasy (*c*_3_)
*m*_4_	Venom (2018)	Horror (*c*_4_)
*m*_5_	The Flash	Science fiction (*c*_5_)

**Table 5:** IDs of sites, movies, user names, and reviews.

ID table					
Site name	Site ID	Movie ID	User name	User ID	Review ID
CinemaBlend	*s*_1_	*m*_1_	Deplorable_me	*u*_1_	*d*_1_
		*m*_2_	Snow gator	*u*_2_	*d*_2_
		*m*_3_	David Curry	*u*_3_	*d*_3_
		*m*_4_	Smedley	*u*_4_	*d*_4_
		*m*_5_	DC villains	*u*_5_	*d*_5_
Moviefone	*s*_2_	*m*_1_	The Guardian Peter Bradshaw	*u*_6_	*d*_6_
		*m*_2_	Snow gator	*u*_7_	*d*_7_
		*m*_3_	Relax ad mike	*u*_8_	*d*_8_
		*m*_4_	Jza Smack	*u*_9_	*d*_9_
		*m*_5_	Clifford De Voe	*u*_10_	*d*_10_
Rotten Tomatoes	*s*_3_	*m*_1_	Jennifer Heaton	*u*_11_	*d*_11_
		*m*_2_	Carlos Díaz Reyes	*u*_12_	*d*_12_
		*m*_3_	Jeffrey Bloomer	*u*_13_	*d*_13_
		*m*_4_	ugene Bernabe	*u*_14_	*d*_14_
		*m*_5_	Dee R.	*u*_15_	*d*_15_

**Table 6:** Semantic emotion of movie reviews of users from CinemaBlend.

Reviews of users about movies				
Movie ID	User name	Review ID	Reviews	Semantic emotion
*m*_1_	*u*_1_	*d*_1_	Unwatchable I made it through 20 minutes I think.	Average Favorable
*m*_2_	*u*_2_	*d*_2_	I did not like it. Thought it was uneven and wasted some great talent. Not funny enough, too much turd humor, and think it is a made for USA level of quality with better effects. It is worth seeing for Blanchett. She does steal every scene, and when the sequel happens—and it has made more than enough money for one—I hope she is front and center as the main character. She and Black do have fantastic chemistry.	Average Favorable
*m*_3_	*u*_3_	*d*_3_	Predator 1 and 2 had comedy in it. Shane Black helped write the original (everyone should know that by now). Predators are the most serious movie of the franchise.	Average Favorable
*m*_4_	*u*_4_	*d*_4_	I went to see it today with open expectations (professional reviews bad, viewer reviews good) and thought it was a fun movie. It cracked me up a couple of times.	Highly Favorable
*m*_5_	*u*_5_	*d*_5_	The Flash has done a fantastic job of incorporating classic from the hero's comic book history	Highly Favorable

**Table 7:** Movie review tokenization and tagging.

Tags		
User ID	Tokens per document	Tagging
*u*_1_	9	Unwatchable/VB I/PRP made/VBD it/PRP through/IN 20/CD minutes/NNS I/PRP think/VBP
*u*_2_	91	Thought/RB it/PRP was/VBD uneven/JJ and/CC wasted/VBD some/DT great/JJ talent/NN ./. Not/RB funny/JJ enough/RB ,/, too/RB much/JJ turd/VBD humor/NN ,/, and/CC think/VBP it/PRP is/VBZ a/DT made/VBN for/IN USA/NNP level/NN of/IN quality/NN with/IN better/JJR effects/NNS ./. It/PRP is/VBZ worth/JJ seeing/VBG for/IN Blanchett/NNP ./.She/PRP does/VBZ steal/VB every/DT scene/NN ,/, and/CC when/WRB the/DT sequel/NN happens/VBZ -/: and/CC it/PRP has/VBZ made/VBN more/RBR than/IN enough/JJ money/NN for/IN one/CD -/: I/PRP hope/VBP she/PRP is/VBZ front/NN and/CC center/NN as/IN the/DT main/JJ character/NN ./. She/PRP and/CC Black/NNP do/VBP have/VB a/DT fantastic/JJ chemistry/NN ./.
*u*_3_	34	Predator/NNP 1/CD &/CC 2/CD had/VBD comedy/NN in/IN it/PRP ./. Shane/NNP Black/NNP helped/VBD write/VB the/DT original/NN -LRB-/-LRB- everyone/NN should/MD know/VB that/DT by/IN now/RB -RRB-/-RRB- ./. Predators/NNS is/VBZ the/DT most/RBS serious/JJ movie/NN of/IN the/DT franchise/NN ./.
*u*_4_	34	I/PRP went/VBD to/TO see/VB it/PRP today/NN with/IN open/JJ expectations/NNS -LRB-/-LRB- professional/JJ reviews/NNS bad/JJ ,/, viewer/CD reviews/NNS good/JJ -RRB-/-RRB- and/CC thought/VBD it/PRP was/VBD a/DT fun/NN movie/NN ./. It/PRP cracked/VBD me/PRP up/IN a/DT couple/NN times/NNS ./.
*u*_5_	17	The/DT Flash/NNP has/VBZ done/VBN a/DT fantastic/JJ job/NN of/IN incorporating/VBG classic/NN from/IN the/DT hero/NN's/POS comic/JJ book/NN history/NN
—	—	—
*u*_*n*_	—	—

**Table 8:** Movie reviews' semantic score.

Aggregated semantic score				
Movie ID	Review ID	CinemaBlend	Moviefone	Rotten Tomatoes
*m*_1_	*d*_1_	0.4	0.6	0.2
*m*_2_	*d*_2_	0.2	0.2	0.4
*m*_3_	*d*_3_	0.2	0.6	0.6
*m*_4_	*d*_4_	0.6	0.2	0.4
*m*_5_	*d*_5_	0.8	0.0	0.0

**Table 9:** Twitter likes.

Twitter likes		
Movie name	Unnormalized	Normalized
*m*_1_	366	366
*m*_2_	154	154
*m*_3_	258	258
*m*_4_	3	3
*m*_5_	2196K	2169

**Table 10:** Movie rating.

Rating						
Movie ID	Unnormalized			Normalized		
	CinemaBlend (%)	Moviefone (%)	Rotten Tomatoes (%)	CinemaBlend	Moviefone	Rotten Tomatoes
*m*_1_	70	16	39	7	1.60	3.90
*m*_2_	80	55	60	4.0	5.50	6.00
*m*_3_	70	25	49	3.5	2.50	4.90
*m*_4_	40	Nil	39	2.0	Nil	3.90
*m*_5_	73	69	93	7.3	6.90	9.30

**Table 11:** Normalized movie votes.

Votes						
Movie ID	Unnormalized			Normalized		
	CinemaBlend	Moviefone	Rotten Tomatoes	CinemaBlend	Moviefone	Rotten Tomatoes
*m*_1_	1.5K	679	4.5K	1500	679	4500
*m*_2_	870	760	6.5K	870	760	6500
*m*_3_	797	890	94	797	890	94
*m*_4_	3.46K	6.9K	910	3460	6900	910
*m*_5_	67	76	8.3K	670	760	8300

**Table 12:** Final score, movie category, medal rank, and genres.

Movie ID	Final score	Genre category	Medal rank	Recommendation of movie
*m*_1_	1.30	Action	Copper	Highly Unpopular
*m*_2_	4.41	Adventure	Silver	Average Popular
*m*_3_	3.63	Fantasy	Bronze	Unpopular
*m*_4_	4.59	Horror	Silver	Average Popular
*m*_5_	4.47	Science fiction	Silver	Average Popular

**Table 13:** Comparison between sentiment classification models.

Classification models	IMDB		Yelp 2013		Yelp 2014	
	Accuracy	RMSE	Accuracy	RMSE	Accuracy	RMSE
*Without using user and product information*						
Majority	0.196	2.495	0.411	1.060	0.392	1.097
Trigram	0.399	1.783	0.569	0.814	0.577	0.804
Text feature	0.402	1.793	0.556	0.845	0.572	0.800
AvgWordvec + SVM	0.304	1.985	0.526	0.898	0.530	0.893
SSWE + SVM	0.312	1.973	0.549	0.849	0.557	0.851
Paragraph vector	0.341	1.814	0.554	0.832	0.564	0.802
RNTN + recurrent	0.400	1.764	0.574	0.804	0.582	0.821
CNN and without UP (UPNN)	0.405	1.629	0.577	0.812	0.585	0.808
NSC	0.443	1.465	0.627	0.701	0.637	0.686
**NSC** **+** **LA**	**0.487**	**1.381**	**0.631**	**0.706**	**0.630**	**0.715**

*Using user and product information*						
Trigram + UPF	0.404	1.764	0.570	0.803	0.576	0.789
Text feature + UPF	0.402	1.774	0.561	1.822	0.579	0.791
JMARS	N/A	1.773	N/A	0.985	N/A	0.999
UPNN (CNN)	0.435	1.602	0.596	0.784	0.608	0.764
UPNN (NSC)	0.471	1.443	0.631	0.702	N/A	N/A
**NSC** **+** **UMA**	**0.533**	**1.281**	**0.650**	**0.692**	**0.667**	**0.654**

**Table 14:** Recommendation model comparisons.

Ref.	NLP/RNN/LSTM	User preferences	NoSQL	Heterogeneous data	Quantitative score (votes, likes, and ratings)	Qualitative score (analysis of reviews)	Multivariates	Multiple data source sites	Popularity medals	Web bot	Categories	User app
[15]	☓	✓	✓	☓	✓	☓	☓	☓	☓	☓	☓	✓
[72]	✓	✓	✓	☓	☓	✓	☓	☓	☓	☓	☓	✓
[73]	✓	✓	☓	☓	☓	✓	☓	☓	☓	☓	☓	☓
[74]	✓	✓	☓	☓	☓	☓	☓	☓	☓	☓	☓	☓
[21]	✓	☓	☓	✓	✓	☓	✓	✓	☓	✓	✓	✓
Proposed work	✓	✓	✓	✓	✓	✓	✓	✓	✓	✓	✓	✓

**Table 15:** Comparisons between different recommendation decision parameters.

Decision parameters	TP	TN	FP	FN
AS	341	237	207	215
WAS	375	355	95	175
AWAS	406	347	116	131
[74]	525	250	90	135
[21]	442	387	114	57
Multivariate system	554	433	8	5

**Table 16:** Results of the experiments.

	AS	WAS	AWAS	[74]	[21]	Multivariate system
Precision	0.6223	0.7979	0.7778	0.8537	0.7950	0.9858
Recall	0.6133	0.6818	0.7561	0.7955	0.8858	0.9911
*F* score	0.6178	0.7353	0.7668	0.8235	0.8379	0.9884
Accuracy	0.5780	0.7300	0.7530	0.7750	0.8290	0.9870

## Data Availability

The data used to support the findings of this study are available from the corresponding author upon request.

## References

[1] Ratnaparkhi K. (2018). *Recommender System for Food in a Restaurant Based on Natural Language Processing and Machine Learning*.

[2] Mukherjee R., Dutta P. S., Sen S. (2001). *Movies2go—A New Approach to Online Movie Recommendation*.

[3] Fasahte U., Gambhir D., Merulingkar M., Monde A., Pokhare A. (2017). Hotel recommendation system, information technology, Atharva College of Engineering, India. *Imperial Journal of Interdisciplinary Research (IJIR)*.

[4] Chawan P. M., Suvarna K., Goyal Y., Thakker J., Bandiwadekar A. (2018). Recommendation system for dining, department of computer engg and info. tech. VJTI, Mumbai, Maharashtra, India. *Journal of Engineering Research and Application*.

[5] Neidhardt J., Kuflik T., Wörndl W. (2018). Special section on recommender systems in tourism. *Information Technology & Tourism*.

[6] Okon E. U., Eke B. O., Asagba P. O. (2018). An improved online book recommender system using collaborative filtering algorithm. *International Journal of Computer Applications*.

[7] Xu J. A., Araki K. An SVM-based personal recommendation system for TV programs.

[8] Baluja S., Seth R., Sivakumar D. Video suggestion and discovery for YouTube: taking random walks through the view graph.

[9] Aalipour E., Ghazisaeedi M. (2017). Recommender system introduction for requests of cancer world. *International Journal of Community Medicine and Public Health*.

[10] Schedl M., Zamani H., Chen C.-W., Deldjoo Y., Elahi M. (2017). Current challenges and visions in music recommender systems research. *International Journal of Multimedia Information Retrieval*.

[11] Casey M.A., Veltkamp R., Goto M., Leman M., Rhodes C., Slaney M. (2008). Content-based music information retrieval: current directions and future challenges. *Proceedings of the IEEE*.

[12] Stan J., Muhlenbach F., Largeron C. (2014). Recommender systems using social network analysis: challenges and future trends. *Encyclopedia of Social Network Analysis and Mining*.

[13] Lam S. K. T., Frankowski D., Riedl J. (2006). *Do You Trust Your Recommendations? An Exploration of Security and Privacy Issues in Recommender Systems*.

[14] Kim K.-R., Lee J.-H., Byeon J.-H., Moon N.-M. (2010). *Recommender System Using the Movie Genre Similarity in Mobile Service*.

[15] Hsieh M.-Y., Chou W.-K., Li K.-C. (2017). Building a mobile movie recommendation service by user rating and APP usage with linked data on Hadoop. *Multimedia Tools and Applications*.

[16] Kim K.-R., Moon N. (2012). Recommender system design using movie genre similarity and preferred genres in SmartPhone. *Multimedia Tools and Applications*.

[17] Gao M., Zhang X. (January 2019). *A movie recommender system from tweets data*.

[18] Badge K., Jyoti Patil (2017). A survey on product recommendation systems using social data and microblogging information. *International Journal of Innovative Research in Computer and Communication Engineering*.

[19] Ishida Y., Uchiya T., Takumi I. (2017). Design and evaluation of a movie recommendation system showing a review for evoking interested. *International Journal of Web Information Systems*.

[20] Vagliano I., Monti D., Morisio M. SemRevRec: a recommender system based on user reviews and linked data.

[21] Ibrahim M., Bajwa I. (2018). Design and application of a multi-variant expert system using apache hadoop framework. *Sustainability*.

[22] Ren F., Quan C. (2012). Linguistic-based emotion analysis and recognition for measuring consumer satisfaction: an application of affective computing. *Information Technology and Management*.

[23] Wakil K., Bakhtyar R., Ali K., Aladdin K. (2015). Improving web movie recommender system based on emotions. *International Journal of Advanced Computer Science and Applications*.

[24] Tom Y., Hazarikaz D., Poria S., Cambria E. (2018). Recent trends in deep learning based natural language processing. https://arxiv.org/pdf/1708.02709.

[25] Postmus S., Bhulai S. (2018). *Recommender System Techniques Applied to Netflix Movie Data*.

[26] Nguyen P. T., Tomeo P., Di Noia T., Di Sciascio E. (2015). Content-based recommendations via DBpedia and Freebase: a case study in the music domain.

[27] Mirizzi R., Di Noia T., Ragone A., Ostuni V. C. (2012). Movie recommendation with DBpedia.

[28] ZhenWu X.-Y. D., Yin C., Huang S., Chen J. (2018). Improving review representations with user attention and product attention for sentiment classification. https://arxiv.org/abs/1801.07861.

[29] Ferreira R., Holanda O., Melo J., Ibert I., Freitas F., Costa E. (2012). *An Agent-Based Semantic Web Blog*.

[30] Cohena W. W., Fan W. (2000). Web-collaborative filtering: recommending music by crawling the Web. *Computer Networks*.

[31] Jose A. V., Jini K. M. (2015). Personalized movie recommender system using rank boosting approach on hadoop. *International Journal for Innovative Research in Science & Technology*.

[32] Godhani G., Dhamecha M. (2018). A study on movie recommendation system using parallel map reduce technology. *International Journal of Engineering Development and Research*.

[33] Li B., Liao Y., Zheng Q. (2014). Precomputed clustering for movie recommendation system in real time. *Journal of Applied Mathematics*.

[34] Zhao L., Lu Z., Jialin S., Pan Q. Y. Matrix factorization+ for movie recommendation.

[35] Nessel J., Cimpa B. The movie oracle-content based movie recommendations.

[36] Sánchez-Moreno D., Ana B., Gil G. (2016). *A Collaborative Filtering Method for Music Recommendation Using Playing Coefficients for Artists and Users*.

[37] Soni K., Goyal R., Vadera B., More S. (2017). A three-way hybrid movie recommendation system. *International Journal of Computer Applications*.

[38] Khalid H., Wu S. (2016). Reducing the cold-start problem by explicit information with mathematical set theory in recommendation systems. *International Journal of Engineering and Computer Science*.

[39] Sarker A., Ginn R., Nikfarjam A. (2015). Utilizing social media data for pharmacovigilance: a review. *Journal of Biomedical Informatics*.

[40] Tumasjan A., Sprenger T. O., Sandner P. G., Welpe I. M. (2010). Predicting elections with Twitter: what 140 characters reveal about political sentiment. *ICWSM*.

[41] He W., Zha S., Li L. (2013). Social media competitive analysis and text mining: a case study in the pizza industry. *International Journal of Information Management*.

[42] Murnane E. L., Counts S. Unraveling abstinence and relapse: smoking cessation reflected in social media.

[43] Diakopoulos N., Naaman M., Kivran-Swaine F. Diamonds in the rough: social media visual analytics for journalistic inquiry.

[44] Baldwin T., Cook P., Lui M., MacKinlay A., Wang L. (2013). *How Noisy Social Media Text, How Different Social Media Sources?*.

[45] Corley C., Cook D., Mikler A., Singh K. (2010). Text and structural data mining of influenza mentions in web and social media. *International Journal of Environmental Research and Public Health*.

[46] Lipton Z. C., Berkowitz J., Elkan C. (2015). A critical review of recurrent neural networks for sequence learning. https://arxiv.org/abs/1506.00019.

[47] Yiny W., Kanny K., Mo Y., Schützey H. (2017). Comparative study of CNN and RNN for natural language processing. https://arxiv.org/abs/1702.01923.

[48] Goldberg D., Nichols D., Oki B. M., Terry D. (1992). Using collaborative filtering to weave an information tapestry. *Communications of the ACM*.

[49] Konstan J. A. (2004). Introduction to recommender systems: algorithms and evaluation. *ACM Transactions on Information Systems*.

[50] Resnick P., Iacovou N., Suchak M., Bergstrom P., Riedl J. GroupLens: an open architecture for collaborative filtering of net news.

[51] Shardanand U., Maes P. Social information filtering: algorithms for automating “word of mouth”.

[52] Chang A., Liao J. F., Chang P. C., Teng C. H., Chen M. H. Application of artificial immune systems combines collaborative filtering in the movie recommendation system.

[53] Reich E. (1983). Users are individuals: individualizing user models. *International Journal of Man-Machine Studies*.

[54] Adomavicius G., Tuzhilin A. (2005). Toward the next generation of recommender systems: a survey of the state-of-the-art and possible extensions. *IEEE Transactions on Knowledge and Data Engineering*.

[55] Min S.-H., Han I. (2005). Detection of the customer time-variant pattern for improving recommender systems. *Expert Systems with Applications*.

[56] Miller B. N., Konstan J. A., Riedl J. (2004). PocketLens. *ACM Transactions on Information Systems*.

[57] Konstan J. A., Miller B. N., Maltz D., Herlocker J. L., Gordon L. R., Riedl J. (1997). Group lens: applying collaborative filtering to usenet news. *Communications of the ACM*.

[58] Cho Y. H., Kim J. K., Kim S. H. (2002). A personalized recommender system based on web usage mining and decision tree induction. *Expert Systems with Applications*.

[59] Schaefer R. Rules for using multi-attribute utility theory for estimating a user’s interests.

[60] Paul M. (2013). *Providing Actionable Recommendations: A Movie Recommendation Algorithm with Explanatory Capability*.

[61] Zhang Y., Zhang M., Liu Y. Incorporating phrase-level sentiment analysis on textual reviews for personalized recommendation.

[62] Bajwa I. S., Iqbal S. (2016). A semi supervised semantic analysis of natural language constraints using Drt and Markov logic. *Science International*.

[63] Su X., Khoshgoftaar T. M. (2009). *A Survey of Collaborative Filtering Techniques*.

[64] Ma Y., Peng H., Cambria E. Targeted aspect-based sentiment analysis via embedding commonsense knowledge into an attentive LSTM.

[65] Hochreiter S., Schmidhuber J. (1997). Long short-term memory. *Neural Computation*.

[66] Bajwa I., Ismail H., Bukhari H., Amin R. (2016). Automated sentiment analysis of natural language text using machine learning. *Sindh University Research Journal-Surj (Science Series)*.

[67] Tang D., Wei F., Yang N., Zhou M., Liu T., Qin B. Learning sentiment-specific word embedding for twitter sentiment classification.

[68] Socher R., Perelygin A., Wu J. Y. Recursive deep models for semantic compositionality over a sentiment treebank.

[69] Ghani U., Bajwa I., Ashfaq A. (2018). A fuzzy logic based intelligent system for measuring customer loyalty and decision making. *Symmetry*.

[70] Yoon K. Convolutional neural networks for sentence classification.

[71] Yang Z., Yang D., Dyer C., He X., Alex Smola, Hovy E. Hierarchical attention networks for document classification.

[72] Wang Y., Wang M., Xu W. (2018). A sentiment-enhanced hybrid recommender system for movie recommendation: a big data analytics framework. *Wireless Communications and Mobile Computing*.

[73] Kumar S., Halder S. S., De K., Roy P. P. Movie recommendation system using sentiment analysis from microblogging data. https://arxiv.org/abs/1811.10804.

[74] Zheng H.-T., Chen J.-Y., Liang N., Sangaiah A. K., Jiang Y., Zhao C.-Z. (2019). A deep temporal neural music recommendation model utilizing music and user metadata. *Applied Science*.

